# MHC-I Affects Infection Intensity but Not Infection Status with a Frequent Avian Malaria Parasite in Blue Tits

**DOI:** 10.1371/journal.pone.0072647

**Published:** 2013-08-30

**Authors:** Helena Westerdahl, Martin Stjernman, Lars Råberg, Mimi Lannefors, Jan-Åke Nilsson

**Affiliations:** Department of Biology, Lund University, Lund, Sweden; Institut Pasteur, France

## Abstract

Host resistance against parasites depends on three aspects: the ability to *prevent*, *control* and *clear* infections. In vertebrates the immune system consists of innate and adaptive immunity. Innate immunity is particularly important for preventing infection and eradicating established infections at an early stage while adaptive immunity is slow, but powerful, and essential for controlling infection intensities and eventually clearing infections. Major Histocompatibility Complex (MHC) molecules are central in adaptive immunity, and studies on parasite resistance and MHC in wild animals have found effects on both infection intensity (parasite load) and infection status (infected or not). It seems MHC can affect both the ability to control infection intensities and the ability to clear infections. However, these two aspects have rarely been considered simultaneously, and their relative importance in natural populations is therefore unclear. Here we investigate if MHC class I genotype affects infection intensity and infection status with a frequent avian malaria infection *Haemoproteus majoris* in a natural population of blue tits *Cyanistes caeruleus*. We found a significant negative association between a single MHC allele and infection intensity but no association with infection status. Blue tits that carry a specific MHC allele seem able to suppress *H. majoris* infection intensity, while we have no evidence that this allele also has an effect on clearance of the *H. majoris* infection, a result that is in contrast with some previous studies of MHC and avian malaria. A likely explanation could be that the clearance rate of avian malaria parasites differs between avian malaria lineages and/or between avian hosts.

## Introduction

Host resistance against parasites depends on three aspects: the ability to prevent infection in the first place, the ability to control infection intensities if the parasite establishes, and the ability to clear the infection. Numerous studies have shown that variation among hosts in each of these three aspects is partly determined by genetics, and a large number of studies have also aimed at determining the more precise molecular genetic basis of the resistance [1]–[4]. In vertebrates, intensively studied genes in association with disease resistance/susceptibility are those found within the Major Histocompatibility Complex (MHC). MHC is a highly polymorphic gene complex, and MHC class I and II molecules are known to play an important role in adaptive immunity [5], [6]. MHC molecules bind peptides and an appropriate immune response should be initiated when the bound peptide is foreign (‘non-self’) while there should be no response when the bound peptide is from the own body (‘self’). Different MHC molecules bind different repertoires of peptides. Laboratory infection experiments have shown that MHC can affect both the ability to control the intensity of an infection [1], [4], [7], [8] and the ability to clear an infection [9].

Several studies have investigated the effects of MHC on host infection status (i.e., presence/absence of infection) in natural populations. Raccoon rabies virus causes acute encephalopathy and exerts a strong selection on raccoons *Procyon lotor* and here MHC class IIB alleles have been found to be associated with infection status [10]. Similarly, host infection status of Puumala virus in bank voles *Myodes glareolus* is associated with specific MHC class IIB alleles [11]. Finally, in songbirds a handful of studies have found associations between infection status of avian malaria and specific MHC class I alleles [12]–[16]. Associations between particular MHC alleles and infection status can be both negative and positive; hence, there seem to be alleles for both resistance and susceptibility [10], [13], [16]. Since the MHC is not directly involved in the initial innate response against a pathogen but only during the slower adaptive response, associations between infection status and specific MHC alleles are most likely the result of effects on the ability to clear the infection, rather than on the ability of the host to completely prevent infection in the first place.

The majority of the studies on MHC in relation to infection intensity in natural host-parasite systems are on intestinal parasites [17]–[21]. Bank voles are frequently infected with intestinal parasites and Kloch et al. (2010) reported an MHC class IIB resistance allele associated with lower infection intensity of a prevalent nematode [18]. More recently studies have begun to investigate the role of MHC in relation to infection intensity also for other infections [22]–[24]. In a natural population of water voles *Arvicola terrestris*, MHC genotype explained the burden of gamasid mites, the flea *Megabothris walker,* and nymphs of sheep ticks *Ixodes ricinus* [22]. However, the effect of MHC on infection status and infection intensity has rarely been considered simultaneously. Hence, there is as yet limited information regarding the relative importance of effects of MHC on control and clearance of infection in natural populations. Furthermore, little is known about whether effects of MHC on the ability to control and clear infections are associated in natural host-parasite systems [25].

In the present study we use the blue tit *Cyanistes caeruleus* and the avian malaria blood parasite *Haemoproteus majoris* to investigate if variation at MHC class I (MHC-I) affects infection intensities and/or infection status, and whether these two effects are congruent. MHC diversity and avian malaria infections constitute a suitable system to study parasite-host interactions in songbirds [26], and both sedentary and migratory passerines are frequently infected with avian malaria, *Haemoproteus sp.* and *Plasmodium sp*. [27], [28]. Passerines that become infected with a novel avian malaria parasite have an initial acute phase of the infection with high infection intensities. Individuals that survive this phase may then carry the parasite as a chronic infection (with lower infection intensities) for long periods of time [29], [30]. Avian malaria (*Haemoproteus sp.* and *Plasmodium sp*.) are vector borne parasites and the main vectors are biting midges and mosquitoes, respectively. Infection experiments and studies from natural populations have shown that infections range from rather mild to very severe and hence avian malaria can have minor to major effects on fitness and survival [29]–[33].

## Methods

### Study Population and Field Work

The blue tits included in this study originate from a nest box population at Revingehed approximately 20 km east of Lund. The birds were caught in 1997–2002 during nestling feeding, ringed and a blood sample was collected. Part of the blood sample was stored in 500 microliter SET buffer (0.15 M NaCl, 0.05 M TRIS, 0.001 M ethylene diamine tetra acetate), frozen (−20°C) until the genomic DNA was isolated using standard phenol/chloroform-isoamylalcohol extraction. Part of the blood sample was used to make a blood smear. 421 adult individuals are included and they were aged as second calendar year birds (2Y) or older (3Y+). Detailed information on fieldwork and collection of life-history data can be found in e.g., [34], [35]. Permits for catching and blood sampling blue tits were approved by the Malmö/Lund Ethical Committee (M126-00, M61-02).

### Malaria Screening Protocol - Parasite Quantification

Blood smears where fixed and stained, and the infection status (infected or non-infected) and infection intensity was determined by light microscopy under 1000×magnification by counting the number of gametocytes (parasites (merozoites) that have invaded a red blood cell and then transformed) of *Haemoproteus* in 10^4^ red blood cells (for details see [35]). Only *Haemoproteus sp* was found in numbers high enough to determine infection intensity. These findings have been confirmed also using PCR-based parasite identification and Sanger sequencing [34].

In our study population of blue tits a single avian malaria infection *Haemoproteus majoris* reaches an overall prevalence of 77%. The prevalence of *H. majoris* increases over time so that older birds are more likely to carry the infection than younger birds, but the infection intensity decreases with age indicating that older birds are better at suppressing the infection [34].

### MHC-I Screening Protocol

Schut et al. (2011) suggested that blue tits have at least 4 MHC-I loci with functional alleles based on Sanger sequencing and Southern Blot and Restriction Fragment Length Polymorphisms (RFLP) [36]. However, a recent study based on Sanger sequencing and Denaturing Gradient Gel Electrophoresis (DGGE) suggests that blue tits have up to 19 MHC-I alleles in total (including non-functional alleles), hence at least 10 loci [37]. Preliminary data from 454-sequencing (NGS) found that blue tits have up to 10 functional MHC class I alleles, hence at least five loci (O’Connor and Westerdahl unpublished data). In the present study we have used primers that were designed to only amplify functional MHC-I genes [36]. We amplify approximately 50% of all the MHC-I alleles in blue tits and avoid amplifying non-functional alleles [36]–[37]. Our primers will not amplify the three nonfunctional alleles previously published (Paca-UA*11, *12 and *13 [37]) since our fw primer does not align here (see alignment Figure S1). These sequence-specific primers (btclassIfw1 3′-TTTACGGCTGTGAYCTCCTGTC-5′; btclassIrv1 5′-CCMTTCYGGGCAGACGTGTTT-3′) target exon 3 and enables a measure of functional MHC-I variation (exon 3 encodes an important part of the peptide binding region of MHC-I molecule) [38]. Several potentially transcribed loci are amplified simultaneously using this single primer combination (transcribed according to [36]). The PCR was run using standard procedures, the PCR products were 205 bp (primers included; the entire exon is 274 bp) and were separated by Reference Strand mediated Conformation Analyses (RSCA; [39]. The RSCA method separates PCR fragment based on base pair composition. Our sequence-specific primer protocol amplified 1–7 exon 3 sequences per individual [40]. We call these exon 3 sequences “MHC-I alleles” although we are aware of that they stem from several loci. The rationale behind this is that in passerine birds, MHC-I alleles often cannot be separated by locus and identical alleles can occur at several loci [41]. Alleles with different base pair compositions are expected to separate well using the RSCA technique and result in RSCA peaks with different RSCA migration distances. However, one RSCA peak could represent more than a single allele, since PCR fragments with similar nucleotide compositions potentially could migrate similar RSCA distances [40]. The RSCA peaks were named according to their RSCA migration distances, MHC-I allele 232–282. We provide a phylogenetic reconstruction based on 67 functional blue tit MHC-I exon 3 sequences and indicate which nucleotide sequences that are representatives of seven of our different RSCA peaks (Figure S2).

### Statistics

In the analyses of associations between MHC alleles and infection intensity, we included MHC alleles that occurred in more than five individuals (16 MHC alleles). We analysed the relationship between presence/absence of different MHC-I alleles and infection intensity using a univariate general linear model (GLM, IBM SPSS Statistics 21); in this analysis only infected individuals were included. Additional factors included in the initial model were age, year, tarsus length, mass, sex and experiment. The infection intensities were log transformed to meet the requirements of parametric tests (ln(parasite intensity+1). Experiments were conducted in our study population of blue tits in 2000, 2001 and 2002 where clutches were reduced or enlarged by 1/3 in a cross-fostering design or kept as controls (for details see [35]). This had an effect on infection intensity [35]. We therefore also included the factor ‘experiment’ in the GLM analyses. Non-significant factors (p>0.05) were excluded from the model. Then as a second step in the model we added all the 16 MHC alleles and let only significant alleles remain. The final model was identical when all MHC alleles were added from the beginning of this analysis.

We then analysed infection status, the relationship between presence/absence of different MHC-I alleles and prevalence of *H. majoris* infection, using a logistic regression (IBM SPSS Statistics 21), initially including above mentioned factors and then MHC alleles that occurred in more than five individuals (17 MHC alleles as more individuals were included in this dataset). The final model was identical when all MHC alleles were added from the beginning of this analysis. We tested for linkage between MHC alleles using cross-tabulation and Pearson’s correlations coefficient applied to binary data for alleles that occurred in frequencies >10% (Janson 1981).

Statistical parameters are only reported for factors included in the final models. All tests are two-tailed and the mean values are given ± standard error (SE). All analyses were conducted using the statistical package IBM SPSS Statistics 21 (SPSS Inc., Chicago, Illinois).

## Results and Discussion

MHC-I genotypes were successfully screened in 421 individuals and in total 21 different MHC-I alleles were detected using our screening protocol designed for functional MHC diversity [36], [40]. Each individual had between one and seven MHC-I alleles (3.87±0.049). We expect to amplify 50% of the MHC-I alleles per individual so the total number of MHC-I alleles (including non-functional alleles) per individual is likely to be approximately twice as high as the number of alleles per individual reported here (Figure S1 and S2; see also [37]). 16 and 17 MHC-I alleles occurred in more than five individuals in infected and in all individuals, respectively. These alleles occurred in frequencies from 0.039 to 0.77 of infected individuals and from 0.019 to 0.77 in all individuals. Only these alleles were included in downstream analyses (Table 1). 77% of the screened individuals were infected with *H. majoris*.

**Table 1:** Frequencies (number of individuals that carry an alleles/total number of individuals) of 21 blue tit MHC-I alleles.

MHC allele	Frequency
220	0.005
232	0.002
**235**	**0.132**
**238**	**0.338**
**240**	**0.173**
**242**	**0.757**
**243**	**0.168**
**245**	**0.165**
**247**	**0.047**
**250**	**0.019**
**253**	**0.050**
**256**	**0.123**
**259**	**0.265**
**262**	**0.468**
**265**	**0.128**
**267**	**0.087**
270	0.012
273	0.012
**276**	**0.097**
**279**	**0.768**
**282**	**0.035**

421 individuals were screened for MHC genotype and 17 MHC alleles that occurred in more than five individuals were included in statistical analyses for infection status (bold). Allele 250 was excluded from the analysis on infection intensity since it only was found in three infected individuals.

**Figure 1:**
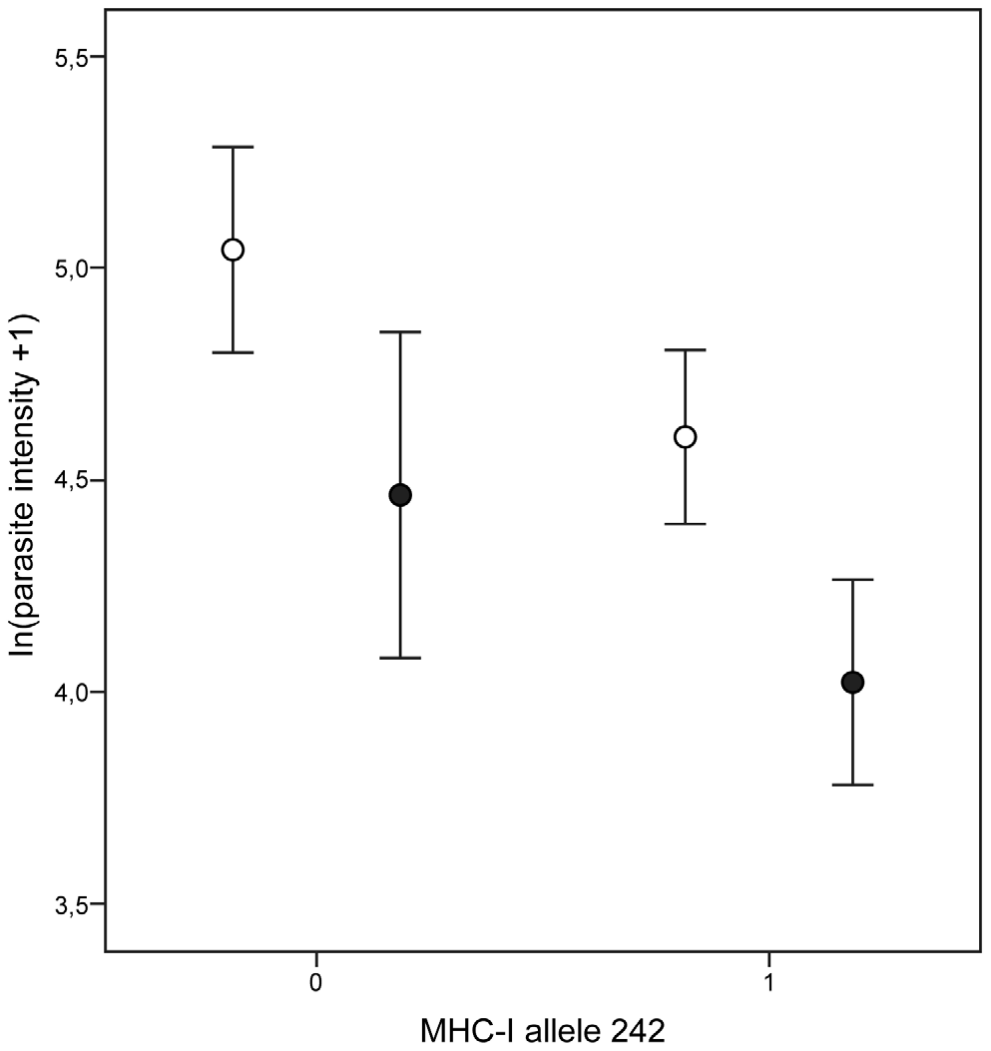
MHC allele 242 suppresses the *H. majoris* infection in both young and old birds. *H. majoris* infection intensity (ln(parasite infection intensity+1)) in blue tits, aged 2Y (open circles) or 3Y+ (filled circles), that carry or do not carry the MHC-I allele 242 (mean±2SE). Allele 242 suppresses the *H. majoris* infection in all birds although young birds (2Y) have higher infection intensities.

Infection intensity was significantly associated with one MHC allele (allele 242) out of the 16 alleles tested (GLM (MHC-I allele 242) *F*_1,301_ = 9.76, p = 0.002, Figure 1). Individuals carrying MHC-I allele 242 had lower infection intensities than individuals without this allele. All individuals with low infection intensities (<0.07% infected red blood cells (ln(parasite infection intensity+1)<2)) carried this allele, while it became comparably less frequent in individuals with higher infection intensities. The infection intensity of the malaria infection *H. majoris* is known to decrease with age [42], and age had a highly significant effect also in the present analysis (GLM (bird age) *F*_1,301_ = 19.1, p<0.001, Figure 1). MHC-I allele 242 suppressed the infection intensity in both young (2Y, one year old) and older birds (3Y+) and there was no interaction between age and allele 242. The brood size manipulation experiment had a close to significant effect on infection intensity with parents in enlarged clutches having higher intensities than control and reduced clutches (GLM (experiment) *F*_1,299_ = 2,84 p = 0.060).

We found no association between infection status with *H. majoris*, and any of the 17 tested MHC-I alleles (p>0.125 in all cases). Thus, we found no evidence that specific MHC-I alleles influence the ability to completely clear the *H. majoris* infection. However, as in previous analyses [34], the probability to become infected with *H. majoris* increased with age (logistic regression, χ^2^ = 9.7, p<0.001). Former studies in passerines have found associations between MHC-I and infection status of avian malaria, in great reed warblers *Acrocephalus arundinaceus*, house sparrows *Passer domesticus* and great tits *Parus major* [12]–[13], [15]–[16], [43]. One difference between our study and these studies is that we screened for prevalence of *Haemoproteus* while the other studies to a large extent have screened for *Plasmodium* (*P. relictum*, SGS1, GRW4, *P.circumflexum* and *P. ashfordi* GRW2. *H. majoris* in blue tits has a much higher prevalence than any of the *Plasmodium* infections in the previously mentioned studies. Blue tits are consequently more likely to become exposed to infection than great reed warblers, house sparrows and great tits. Perhaps the higher risk of being infected results in different immunological strategies, for example, it may be more costly for blue tits to completely clear a common infection than to tolerate low or moderate infection intensities.

Avian malaria infections typically consist of an initial short acute phase with a high parasite load, and a subsequent long chronic phase with lower parasite load [31]. The vast majority of our samples of infected blue tits are presumably from the chronic phase [34]. There is limited knowledge on the rate of clearance of acute phase avian *Plasmodum* infections and even less from *Haemoproteus* infections [29]–[32]. Prior work from Stjernman (2004) suggests that blue tits in our study population once screened as infected with *H. majoris* (during the chronic phase infection) do not clear the infection [42]. Given that there is no clearance of the chronic infection, the uninfected individuals include three different categories of individuals; those that never where exposed to the infection, those that have mounted a successful innate immune response that prevented the infection to be established in the first place and those that completely cleared the acute phase infection. Consequently, as the MHC can only play a role in the latter, a potential reason for why we do not find any association between *H. majoris* infection status and MHC allele 242 in the blue tits could be that blue tits rarely clear the acute phase infection.

What proportion of the variation in infection intensity among individuals can we expect to be explained by MHC? A recent Genome Wide Association Study in 2500 HIV-infected Caucasian men was powered to detect allelic variants that explained >1.3% of the variation in infection intensity. MHC was the genetic region that explained most of the variation in infection intensity and two different MHC alleles explained five and six percent of the variation [44]. In our study on blue tits, the sample size is nearly an order of magnitude lower than in the human HIV study, thus we do not have the power to detect very small differences in *H. majoris* infection intensity. We did, however, find a significant effect of a single MHC-I allele and it explained 2.5% of the variation in *H. majoris* infection intensity (from the GLM (MHC allele 242); r^2^ = 0.025). Thus, in both the human HIV study and our blue tit study, specific MHC alleles explained variation in infection intensity to a similar degree.

MHC is a tightly linked gene complex and several alleles are therefore often found to be inherited as single haplotypes. If alleles are positively associated this can cause statistical problems (see [45] for discussion) and therefore we analyzed associations between eleven blue tit MHC alleles that occurred in at least 10% of the individuals. Eight out of 55 pairwise tests (11×11 alleles) for associations between MHC alleles had p-values <0.05 and all were negative (φ-coefficients between −0.414 and −0.133; Table S1). So, there was no indication that positive associations among alleles confounded our results. Four out of the eight negative associations were significant after Bonferroni correction (at p<0.0045); MHC allele 242 was negatively associated with allele 243 (φ = −0.414, p<0.001), allele 240 (φ = −0.341, p<0.001) and allele 238 (φ = −0.176, p = 0.002), hence individuals that carried allele 242 were less likely to also carry alleles 243, 240 and 238. This could be an effect of that these four alleles are found at the same locus, and when allele 242 is selected for the three others are simultaneously selected against. However, one individual carried all four alleles (238, 240 242 and 243), and nine individuals carried three of these alleles, suggesting that these four alleles must be found at at least two different loci. Finally, allele 256 was negatively associated with allele 259 (φ = −0.185, p = 0.001, Table S1).

## Conclusions

We have investigated if MHC-I alleles could explain infection intensity and infection status of an avian malaria infection (*H. majoris*) in a natural population of blue tits. One MHC allele (allele 242) was significantly associated with lower infection intensities, but no MHC allele was associated with infection status. The blue tits in our study population seem unable to clear a *H. majoris* infection once it has reached the chronic phase, which could explain why we did not find any association with infection status. We imagine that MHC alleles for disease resistance that control the intensity of rather mild infections, like *H. majoris* in blue tits, are unlikely to be associated with infection status when there is no clearance of the infection.

## Supporting Information

Figure S1 Alignment of blue tit MHC-I exon 3 nucleotide sequences (species specific nomenclature Paca and Cyca, and GenBank accession numbers; Paca-UA*1–13 AM232705–AM232717; Paca-UA*101–117, JF742764–80; Cyca-UA*14–53, HQ393911–HQ393951), in comparison with our primers that were designed to preferentially amplify functional alleles. The reverse primer ‘btclassIrv1’ amplify 35 out of 70 available blue tit MHC-I sequences and the forward primer ‘btclassIfw1’ does not amplify the non-functional alleles Paca-UA*11–13. Identity with sequence Paca-UA*8 is indicated with dots. The alleles Paca-UA*107, 102, 109, 114, 108, 117 and 104 (in larger font) correspond to seven different RSCA peaks and are arranged in the alignment according to their RSCA migration distances (Paca-UA*107 migrates the shortest distance and Paca-UA*104 the longest) [36], [40].
(PDF)

Figure S2 Phylogenetic reconstruction of blue tit MHC-I exon 3 nucleotide sequences (species specific nomenclature Paca and Cyca, and GenBank accession numbers; Paca-UA*1–13, AM232705–AM232717; Paca-UA*101–117, JF742764–80; Cyca-UA*14–53, HQ393911–HQ393951), with great reed warbler as outgroup (AcarcN15, AJ005505) using Neighbor-joining (Kimura-2-parameter model, bootstrap (bt) values based on 2000 replicates) **[36]**, **[37]** **.** There are two significant clusters with more than ten alleles (bt = 95 and bt = 99) and one additional cluster with less support (bt = 57). The deeper nodes in the tree are not resolved and 22 alleles are found outside these three clusters. Our primers amplify the alleles outside these three clusters and also the alleles within the cluster with bt = 99. Alleles that potentially are amplified with our primers are indicated with diamonds, and filled diamonds indicate alleles that correspond to specific RSCA peaks [40].
(PDF)

Table S1 Pair-wise correlation between eleven MHC-I alleles occurring in >10% of the individuals using Pearson’s correlations coefficient applied to binary data and Fisher exact for significance level (phi-coefficient, exact p-values or p<0.001).
(DOCX)
