# Supplementary material for: MHC-I Affects Infection Intensity but Not Infection Status with a Frequent Avian Malaria Parasite in Blue Tits
Source: PLoS One. 2013 Aug 30;8(8):e72647. doi: 10.1371/journal.pone.0072647 (PMC3758318; doi:10.1371/journal.pone.0072647)
Supplement: Figure S1 — Alignment of blue tit MHC-I exon 3 nucleotide sequences (species specific nomenclature Paca and Cyca, and GenBank accession numbers; Paca-UA*1–13 AM232705–AM232717; Paca-UA*101–117, JF742764–80; Cyca-UA*14–53, HQ393911–HQ393951), in comparison with our primers that were designed to preferentially amplify functional alleles. The reverse primer ‘btclassIrv1’ amplify 35 out of 70 available blue tit MHC-I sequences and the forward primer ‘btclassIfw1’ does not amplify the non-functional alleles Paca-UA*11–13. Identity with sequence Paca-UA*8 is indicated with dots. The alleles Paca-UA*107, 102, 109, 114, 108, 117 and 104 (in larger font) correspond to seven different RSCA peaks and are arranged in the alignment according to their RSCA migration distances (Paca-UA*107 migrates the shortest distance and Paca-UA*104 the longest) [36], [40]. (PDF) [file pone.0072647.s001.pdf]

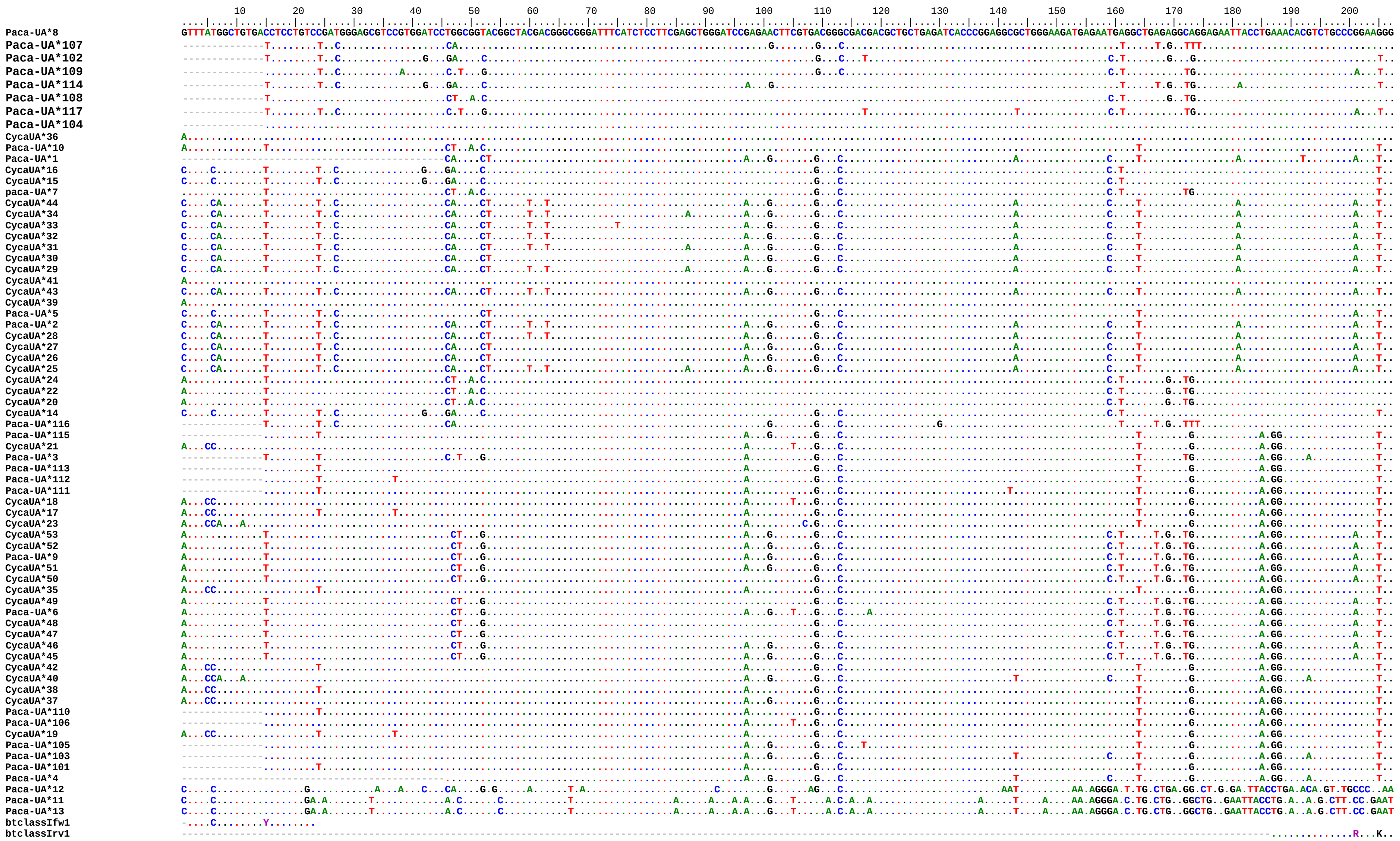
10 20 30 40 50 60 70 80 90 100 110 120 130 140 150 160 170 180 190 200
Paca-UA*8
Paca-UA*107
Paca-UA*102
Paca-UA*109
Paca-UA*114
Paca-UA*108
Paca-UA*117
Paca-UA*104
CycaUA*36
Paca-UA*10
Paca-UA*1
CycaUA*16
CycaUA*15
paca-UA*7
CycaUA*44
CycaUA*34
CycaUA*33
CycaUA*32
CycaUA*31
CycaUA*30
CycaUA*29
CycaUA*41
CycaUA*43
CycaUA*39
Paca-UA*5
Paca-UA*2
CycaUA*28
CycaUA*27
CycaUA*26
CycaUA*25
CycaUA*24
CycaUA*22
CycaUA*20
CycaUA*14
Paca-UA*116
Paca-UA*115
CycaUA*21
Paca-UA*3
Paca-UA*113
Paca-UA*112
Paca-UA*111
CycaUA*18
CycaUA*17
CycaUA*23
CycaUA*53
CycaUA*52
Paca-UA*9
CycaUA*51
CycaUA*50
CycaUA*35
CycaUA*49
Paca-UA*6
CycaUA*48
CycaUA*47
CycaUA*46
CycaUA*45
CycaUA*42
CycaUA*40
CycaUA*38
CycaUA*37
Paca-UA*110
Paca-UA*106
CycaUA*19
Paca-UA*105
Paca-UA*103
Paca-UA*101
Paca-UA*4
Paca-UA*12
Paca-UA*11
Paca-UA*13
btclassIfw1
btclassIrv1
