# Supplementary figures and images for: MHC-I Affects Infection Intensity but Not Infection Status with a Frequent Avian Malaria Parasite in Blue Tits
Source: PLoS One. 2013 Aug 30;8(8):e72647. doi: 10.1371/journal.pone.0072647 (PMC3758318; doi:10.1371/journal.pone.0072647)

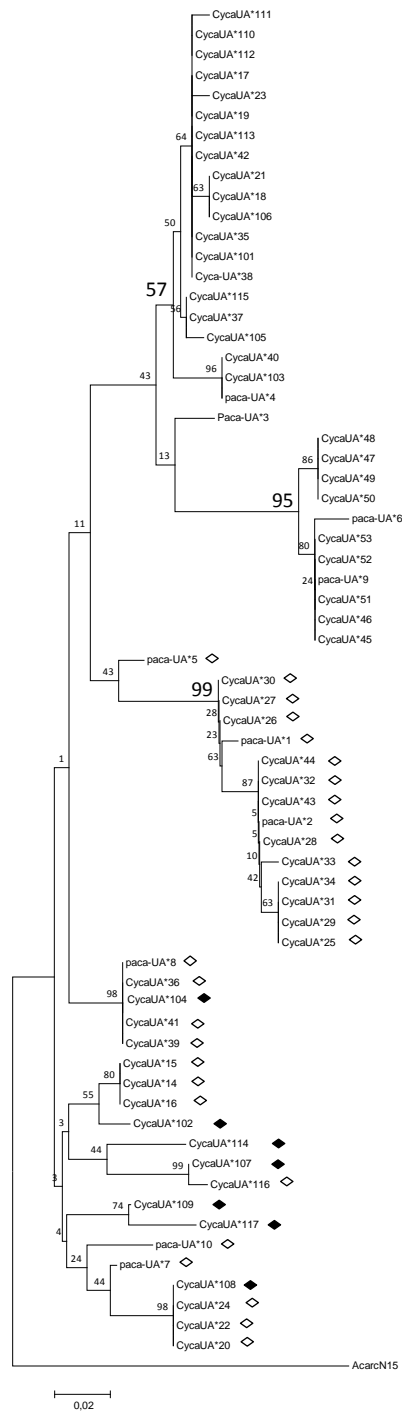

Supplement: Figure S2 — Phylogenetic reconstruction of blue tit MHC-I exon 3 nucleotide sequences (species specific nomenclature Paca and Cyca, and GenBank accession numbers; Paca-UA*1–13, AM232705–AM232717; Paca-UA*101–117, JF742764–80; Cyca-UA*14–53, HQ393911–HQ393951), with great reed warbler as outgroup (AcarcN15, AJ005505) using Neighbor-joining (Kimura-2-parameter model, bootstrap (bt) values based on 2000 replicates) [36] , [37] . There are two significant clusters with more than ten alleles (bt = 95 and bt = 99) and one additional cluster with less support (bt = 57). The deeper nodes in the tree are not resolved and 22 alleles are found outside these three clusters. Our primers amplify the alleles outside these three clusters and also the alleles within the cluster with bt = 99. Alleles that potentially are amplified with our primers are indicated with diamonds, and filled diamonds indicate alleles that correspond to specific RSCA peaks [40]. (PDF) [file pone.0072647.s002.pdf]
