# Supplementary material for: MHC-I Affects Infection Intensity but Not Infection Status with a Frequent Avian Malaria Parasite in Blue Tits
Source: PLoS One. 2013 Aug 30;8(8):e72647. doi: 10.1371/journal.pone.0072647 (PMC3758318; doi:10.1371/journal.pone.0072647)
Supplement: Table S1 — Pair-wise correlation between eleven MHC-I alleles occurring in >10% of the individuals using Pearson’s correlations coefficient applied to binary data and Fisher exact for significance level (phi-coefficient, exact p-values or p<0.001). (DOCX) [file pone.0072647.s003.docx]

**Table S1.** Pair-wise correlation between eleven MHC-I alleles occurring in >10% of the individuals using Pearson’s correlations coefficient applied to binary data and Fisher exact for significance level (phi-coefficient, exact p-values or p<0.001).

| **allele** | **235** | **238** | **240** | **242** | **243** | **245** | **256** | **259** | **262** | **265** | **279** |
| --- | --- | --- | --- | --- | --- | --- | --- | --- | --- | --- | --- |
| **235** | X | -0,074 | 0,0363 | -0,093 | -0,057 | -0,033 | -0,06 | -0,014 | -0,077 | -0,089 | -0,09 |
| **238** |  | X | -0,101 | **-0,176, p=0.002** | 0,0517 | 0,0498 | 0,0969 | -0,046 | -0,079 | -0,088 | 0,0795 |
| **240** |  |  | X | **-0,341, p<0.001** | -0,098 | 0,0678 | -0,133, p=0.019 | -0,102 | 0,0417 | 0,0663 | 0,0234 |
| **242** |  |  |  | X | **-0,414, p<0.001** | -0,149, p=0.009 | -0,031 | 0,0892 | 0,0004 | 0,0594 | -0,041 |
| **243** |  |  |  |  | X | 0,076 | 0,0483 | -0,036 | -0,093 | -0,002 | -0,295 |
| **245** |  |  |  |  |  | X | -0,029 | -0,036 | -0,014 | -0,002 | -0,087 |
| **256** |  |  |  |  |  |  | X | **-0,185, p=0.001** | -0,107 | -0,145, p=0.011 | -0,073 |
| **259** |  |  |  |  |  |  |  | X | 0,0166 | -0,053 | 0,0988 |
| **262** |  |  |  |  |  |  |  |  | X | -0,146, p=0.01 | 0,0028 |
| **265** |  |  |  |  |  |  |  |  |  | X | 0,0434 |
| **279** |  |  |  |  |  |  |  |  |  |  | X |
